# A reduced-dimensionality approach to uncovering dyadic modes of body motion in conversations

**DOI:** 10.1371/journal.pone.0170786

**Published:** 2017-01-31

**Authors:** Guy Gaziv, Lior Noy, Yuvalal Liron, Uri Alon

**Affiliations:** 1 Department of Molecular Cell Biology, Weizmann Institute of Science, Rehovot, Israel; 2 The Theatre Lab, Weizmann Institute of Science, Rehovot, Israel; Ghent University, BELGIUM

## Abstract

Face-to-face conversations are central to human communication and a fascinating example of joint action. Beyond verbal content, one of the primary ways in which information is conveyed in conversations is body language. Body motion in natural conversations has been difficult to study precisely due to the large number of coordinates at play. There is need for fresh approaches to analyze and understand the data, in order to ask whether dyads show basic building blocks of coupled motion. Here we present a method for analyzing body motion during joint action using depth-sensing cameras, and use it to analyze a sample of scientific conversations. Our method consists of three steps: defining modes of body motion of individual participants, defining dyadic modes made of combinations of these individual modes, and lastly defining motion motifs as dyadic modes that occur significantly more often than expected given the single-person motion statistics. As a proof-of-concept, we analyze the motion of 12 dyads of scientists measured using two Microsoft Kinect cameras. In our sample, we find that out of many possible modes, only two were motion motifs: synchronized parallel torso motion in which the participants swayed from side to side in sync, and still segments where neither person moved. We find evidence of dyad individuality in the use of motion modes. For a randomly selected subset of 5 dyads, this individuality was maintained for at least 6 months. The present approach to simplify complex motion data and to define motion motifs may be used to understand other joint tasks and interactions. The analysis tools developed here and the motion dataset are publicly available.

## Introduction

Understanding joint human action is of great interest. During joint action people move in complex and coordinated ways to achieve shared goals [1, 2]. Joint action [3, 4] has been mostly studied in the context of defined laboratory tasks, designed to reduce the motion to one or a few degrees of freedom which can be fruitfully analyzed [5–15]. However, joint action in natural settings involves tens of three-dimensional degrees of freedom per person. To deal with such complexities, studying joint action in naturalistic settings requires new methodologies. Here we outline commonly employed methodologies and propose a new method to analyze spatial modes of dyadic motion during naturalistic joint action. We demonstrate a proof-of-concept for this methodology on a sample of dyads engaged in scientific conversations.

### Methods for studying joint action in simple tasks

Because of the complexity of human motion, many studies of joint action employ special tasks, characterized by lower dimensionality when compared with naturalistic tasks. Examples of such tasks include tapping fingers [9], rocking chairs [7], swinging penduli [5, 16], stepping together [11] and the mirror game [12, 14]. The 1D signal in these tasks (e.g. the timing of the tapping fingers, the relative phase of the penduli etc.) simplifies the analysis.

This simplicity enabled discoveries about the way people perform tasks together. For example, Oullier et al. used a controlled paradigm in which humans unintentionally synchronize their motion by merely exchanging visual information while tapping their finger. The inclusion of visual information exchange was sufficient to induce frequency and phase matching of the tapping [9].

Such controlled experiments lend themselves to mathematical analysis. Theory has been developed which maps ideas from inter-limb coordination to the coordination between people [17, 18]. The most widely used theory is the Haken-Kelso-Bunz model [19]. Such dynamical theory offers predictions which have been experimentally tested, such as critical phenomena in which anti-phase synchrony becomes destabilized at high frequencies.

Recently, a fascinating line of work on interpersonal motion synergies expanded the scope of joint action analyses beyond one degree of freedom [13, 16, 20, 21]. Studies of motion synergies are inspired by Bernstein’s degree of freedom problem, which suggests that many degrees of freedom must couple to reduce the computational burden placed on the nervous system [22]. Motion synergy is defined by low dimensionality of the data together with reciprocal compensation in which the two actors compensate for each other’s variations along the coordinates relevant to the task [20]. Dimensional compression and reciprocal compensation can be measured by principal component analysis and the uncontrolled manifold (UCM) approach respectively [16, 20]. One drawback of the UCM approach is that it requires defining a performance axis relative to which the compensation is defined. Such an axis may be difficult to define a-priori in naturalistic tasks such as conversations.

Synergistic aspects of joint action have been suggested to provide a better statistical predictor of collective performance than synchrony [20, 23, 24]. For example, Abney et al. showed that dyads engaged in a joint task performed better when they exhibited weak coupling of body movement. They suggested that simple synchrony may not be the key to effective performance [25]. As focus on synergy progresses, so does the need for new methodologies that introduce analyses of higher complexity.

### Methods for studying naturalistic joint action

In addition to studying simple tasks, researchers have been studying naturalistic joint action for decades. These tasks are essentially complex, and involve many degrees of freedom.

Early work employed manual coding of videos [26–30]. Outstanding examples include Condon and Sander’s work on the synchrony of speech and body motion, Newtson’s coding of motion according to the Sharet-Wachtel method, and widely used coding methods by Bernieri et al. [31–33]. Temporal patterns of dyadic movement elements can be analyzed in this way, such as T-patterns [34, 35].

Manual coding has been extensively used to explore conversations. Kendon showed how people arrange in particular spatial formations during conversation, with defined protocols for entry and exit into the space defined by the participants [36]. Other manual studies of conversations explored the use of back-channels [37–40], interactive alignment [24, 41–53], gaze direction and turn taking [53–59]. Such coding has been routinely used in social and developmental psychology, but has the limitation of being labor intensive, limiting the scope of possible studies.

More recently, gains in efficiency and precision were obtained by automated analyses of naturalistic interactions. Some approaches reduce the motion as captured in video to one dimensional movement signals. One commonly-used signal is the total motion energy of each of the interactants. Motion energy for each person is computed by the difference between video frames cropped to include that person (frame differencing method, FDM, or optical flow [25, 60–64]).

These automated methods have been used to study interpersonal synchrony and its correlates under diverse natural settings. Such settings include psychotherapy sessions [60, 61, 65], friendly conversations [62], conversations where interactants tell each other knock-knock jokes [63], joint food-tower construction [25], and interactions between doctors and patients [14]. In these studies the authors established links between interpersonal synchrony and conversation content [66], mutual rapport [62], and relationship quality [60, 61, 65].

Automated image analysis has also been used for more than two people, as in the case of sports. Representing each player as a point, researchers have studied group phase using models for synchronization of multiple oscillators [67]. Other approaches of automated analysis use tracking systems. For example Shockley et al. used pressure plates to track conversants’ body sway. They showed postural entrainment when people describe pictures to each other [68, 69].

Recent technological advances in depth cameras and motion capture system provide easy access to quantitative high-dimensional data from conversations. However, most analysis methods for this data remain one dimensional. For example, Won et al. used Kinect depth cameras that provide 3D motion for skeletal joints. They showed that certain coordinates tended to synchronize between people and that overall synchrony correlated with creativity in defined tasks [70]. To cope with the high dimensionality of the data, multiple 1D analyses were performed, each targeting specific combinations of joints’ motion in the dyad.

Other researchers acquired 3D data of head and hand motion during natural conversations using advanced motion capture systems [71, 72]. To analyze this data, Boker et al. used a 1D analysis approach named windowed cross-correlation. They found that the prevalence of gaze and hand coordination increases with the loudness of the environment, so as to disambiguate the conversants’ verbal communication [71]. Using qualitatively similar data, Lavelle et al. performed a 1D analysis to show that nonverbal communication, including head nods and hand gestures, is disrupted in conversations involving patients with schizophrenia [72]. In both of these examples, the spatial nature of the acquired data was reduced to 1D by averaging or selecting coordinates of interest.

**Fig 1:**
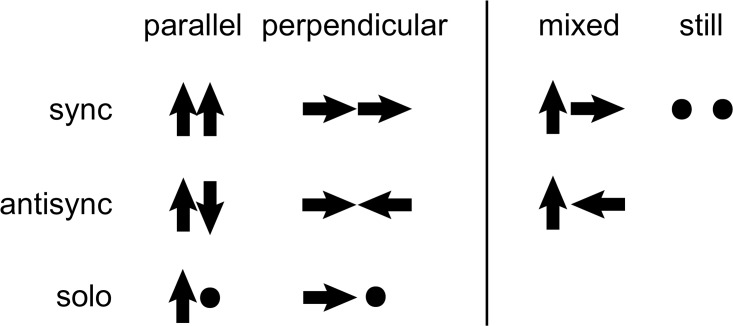
Eight possible modes of dyadic torso motion. Each mode has either parallel motion (vertical arrow), perpendicular motion (horizontal arrow) or stillness (dot) for each of the two conversants. We consider the two mixed modes as a single mode in our analysis. Directions refer to motion relative to participant’s own pelvis.

In order to advance beyond 1D under naturalistic settings requires addressing the large possible number of spatial motion modes. For example, consider two people facing each other. Suppose for simplicity that each person can sway parallel or perpendicular to the torso, or be still. Thus each person has three ‘motion modes’. These can combine interpersonally to form at least 8 possible spatial dyadic modes of motion (both sway parallel in sync, both sway parallel in anti-sync, one sways parallel and the other perpendicular etc., Fig 1).

Here we develop a method to detect which of these dyadic spatial modes occur in a given interaction, and to test whether they appear due to the dyadic interaction, namely beyond their expected representation by random motion correlations. The method involves three steps: defining individual modes, defining dyadic modes made of combinations of these individual modes, and defining motion motifs which are dyadic modes that occur more often than chance. We demonstrate the method on a sample of 12 dyads having scientific conversations measured by depth cameras. This small sample is intended as an example application of the method rather than as a basis for theoretical statements on the results. The full motion dataset and video data are available online in the Dryad digital repository (doi:10.5061/dryad.804j3). We also provide open-source software of the analysis tools for acquiring the data from two cameras and performing the data analysis steps including motion motif analysis (https://github.com/ggaziv/DualKinect).

## Materials and Methods

### Participants

12 dyads of graduate students (*n* = 20), postdocs (*n* = 1), or faculty (*n* = 3) in physics, biology, or computer science [age mean (SEM) 31.8 (1.15)] participated in this study. All dyads but one were pairs who were familiar with each other prior to experiment and had had numerous scientific conversations with each other. Dyadic gender was male-male (*n* = 9), female-female (*n* = 1), and mixed (*n* = 2). Academic positions were student-student (*n* = 9), postdoc-student (*n* = 1), faculty-faculty (*n* = 1), and faculty-student (*n* = 1). The research protocol was reviewed and approved by the Bioethics and Embryonic Stem Cell Research Oversight (ESCRO) Committee at the Weizmann Institute of Science. Participants gave their written informed consent to be recorded, and were not granted incentives for their participation.

### Setup

**Fig 2:**
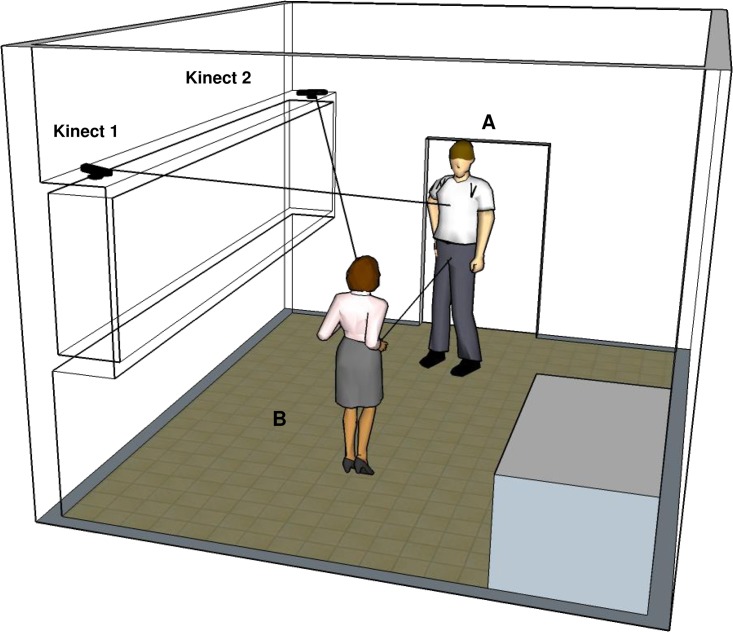
Experimental setup schematics. Two cameras are directed toward their corresponding participants in the room.

**Fig 3:**
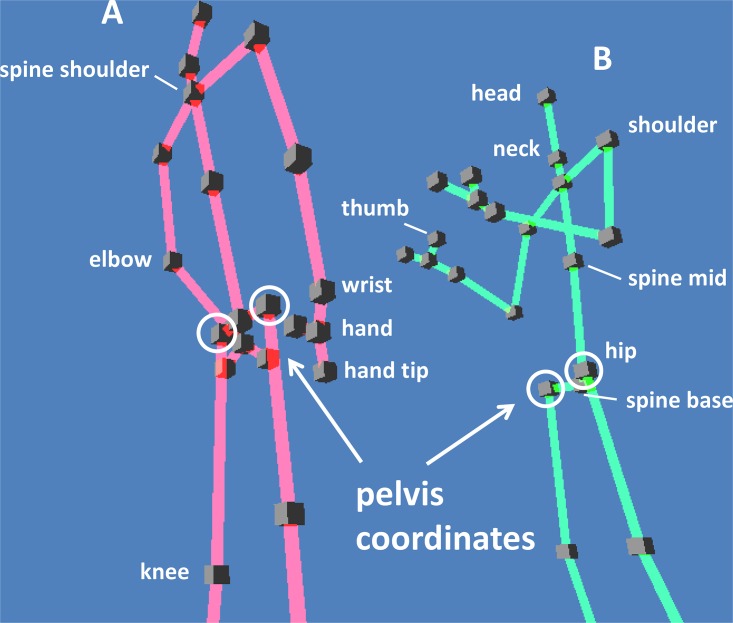
Illustration of dyad skeletal features measured in the experiment.

Two Microsoft Kinect^™^ depth-sensing cameras were positioned in a typical scientists’ office (5 sq m) at a height of 2m at a distance of about 180cm from target participant (Fig 2). Distance between the cameras was 220cm. We connected each camera to a different, dedicated computer, and used Kinect Studio v2.0 for data acquisition. We recorded the 3D positions of 25 skeletal joints (Fig 3). In addition, we recorded video and sound of the session with a standard video camera.

### Procedure

#### Preparations prior to experiment

We invited participants to take part in a recorded dialogue session aiming to study nonverbal communication in scientific conversations. We asked them prior to the experiment to share with us names of colleagues with whom they often engage in scientific discussions and feel comfortable, and matched the dyads accordingly.

#### Running the experiment

We aimed to allow natural conversations. We designed the experiment to provide conditions in which one person leads, then the other, and then a condition with mutual leadership [12]. We told the participants that the recorded session consists of 3 rounds of flexible duration between 10-15min each. We asked that on the first two rounds they “consult about a scientific experience or share a significant moment of their scientific work”, led in each round by one of them. Participants transitioned from round one to round two on their own their own pace. Once both rounds were over, the participants called the experimenter, who was waiting in an adjacent room, to provide a joint matter for discussion for the third round. The matter for discussion was one of the following: (1) discuss and rate together the 3 most important scientific or technological breakthroughs anticipated in the next 20 years, or (2) discuss and rate together the 3 most important things which should be taught at school.

We asked participants to behave as they normally would during the conversations, and to converse on matters that they sincerely like or want to talk about. We also asked participants to mind the experimental setup in several regards: to avoid obstruction of the cameras, avoid cornering of one participant to either side of the room, avoid position-switching with their partner, and refrain from using the whiteboard. At the end of the session, participants were asked about their subjective experience throughout the session. Participants were not provided with additional information regarding our analyses beyond the originally declared intent of studying nonverbal behavior in conversations.

We tested these instructions in a pilot experiment and received feedback that the conversations were perceived by participants to be natural and realistic.

#### Additional experiments

Six months after the first experimental rounds, we invited five of the dyads to participate in an additional session. The recording session consisted of a single round. The round had the format of the previous third round, with the alternate joint matter for discussion relative to the one they addressed previously. Same rules and instructions as in the first session were applied.

## Data Analysis

As raw data, we used the Kinect Studio v2.0 software data stream with joint position data for 25 skeletal joints at a baseline resolution of 512x424 pixels, depth resolution at the millimeter scale, and 30fps. The joints include head, torso, shoulders, arms, hands, hips, and feet (Fig 3). Although motion was smooth, the tracking system introduced errors due in part to interference between the cameras. About 70% of the frames had at least one joint in the upper body which was not fully tracked according to Kinect software. To smooth the data we removed position points which deviate discontinuously from their neighbors using a filter that removes the extreme points in a 5-timepoint window before averaging the remaining points. We then used three consecutive rounds of a 5-window median filter. After this smoothing, the data is suitable for differentiation to obtain velocities. Finally, for visualization purposes we employed a square-pulse low pass filter at 3Hz (which was not used for the analysis steps).

We chose to analyze velocity rather than position, because previous studies suggest that people register velocity synchronization rather than position as a primary signal [73, 74]. Velocity allows a translationally invariant analysis: if one participant moves a step in a certain direction, and then continues the same motion, displacement analysis looks different whereas velocity analysis (after the step) looks identical. Similar findings were found in analyzing joint improvisation using the mirror game [12]. The current measurements are accurate enough such that taking the derivative to compute velocity does not introduce noise that is too large for our purpose. We provide an analysis of the data using displacement in the SI section 5 and 6.

### Individual PCA

We considered the speed (root of the sum of the squared x, y and z velocities) of each of 13 upper body joints per participant for time samples acquired throughout the three-round dataset. We normalized the speed of each joint to mean zero and standard deviation of one, and performed principal component analysis using Matlab r2015b. To determine the significant principal components (PCs), we considered a shuffled version of the data where the speed vector for each joint was shuffled. Significant PCs were determined to be those whose explained variance in the real data exceeded by 3 SDs the explained variance of the corresponding PCs from 1000 shuffled datasets [75].

### Determining Still and Solo Motion Segments

Using a sliding window of 2-sec, we calculated the RMS of this speed in each window for each participant. We then compared it with two corresponding thresholds to mark the segment as ‘still’, ‘solo’ of either party, or ‘co-active’. The threshold used for each participant, and each round was the maximum of: (i) 15% max speed of the participant in the round and (ii) 15% max speed of all participants in the entire dataset. We classified ‘solo’ segments to either parallel or perpendicular solo by comparing the RMS of parallel and perpendicular speeds in the segments.

### Dyadic Segment-wise PCA

Similar to Boker et al., we hypothesized that intervals of interpersonal interaction occur within time windows of a few seconds [71]. We employed a sliding window of 2-sec to segment the time series of each round of conversation. In our sample, PCA on the dyadic motion was dominated by still and solo motion. In order to detect co-active principal components, we separated out only the co-active segments. To find the prominent modes in co-active segments we computed PCA on each of them and marked the first PC as the mode represented in the middle point of this segment.

### Parallel Mode Distributions

**Fig 4:**
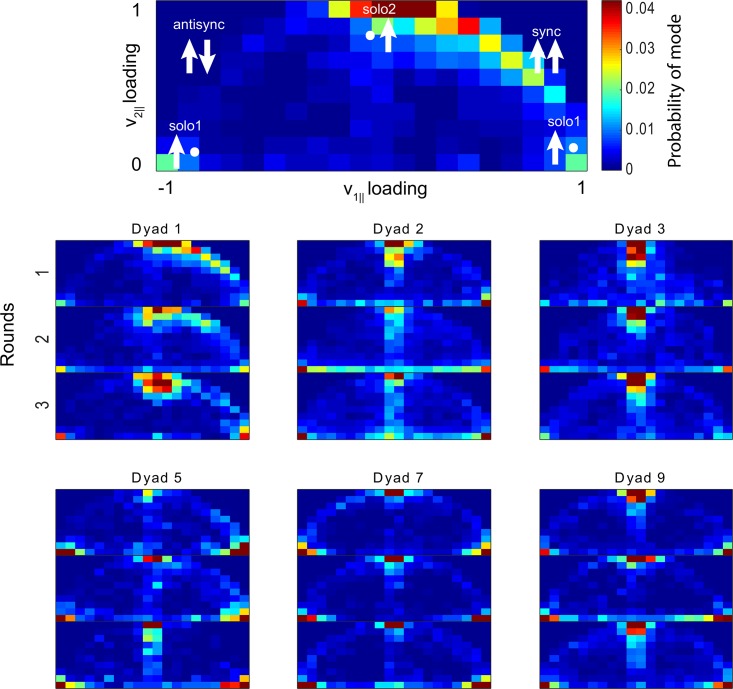
Motion mode probability distribution function for six dyads across the three rounds. Axes are the parallel and perpendicular torso velocity loadings of the first PC in each 2-sec time window, with the first PC normalized to length one. Red colors indicate high probability and blue colors indicate low probability. Pure modes correspond to specific points in the 2D histograms indicated by white arrows. In our sample, dyads exhibit individuality: their distributions are more similar to themselves across rounds than to other dyads.

To visualize the modes we plotted their projection on the two parallel loadings from segment-wise PCA data (Fig 4). Since principal components are only determined up to their sign, we flipped signs of both loadings as needed in order to place the distribution into the upper half plane. Still motion segments were discarded in these plots. To quantitatively compare between distributions, we used the RMS of their bin-wise differences as a distance metric.

### Classification of Torso Modes to Pure Modes

We used a linear classifier to map co-active modes to pure modes (Fig 1). Pure modes are projections of co-active modes onto a pure modes space. We denote this space as $span({\left\{\bar{p_{m}}\right\}}_{m})$, where $\bar{p_{m}},\;p_{m,i}\in \{\pm 1/\sqrt{2},0\}$ denotes the co-active pure mode *m*, characterized by 4 loadings, parallel and perpendicular for each participant. This projection yields scores of classification to each of the pure modes:

$$Score\left(\bar{p_{m}}\right)=\left|\bar{p_{m}}\cdot {\left(\begin{array}{cccc} v_{1||} & v_{1+} & v_{2||} & v_{2+} \end{array}\right)}^{T}\right|.$$

Maximal score determines the co-active pure mode in the segment.

### Defining Motion Motifs

To detect motion motifs, we prepared a control dataset by cyclically shifting the time axis of one of the two participants by at least one minute. We repeated the motion-mode analysis on M = 30 shifted datasets to obtain a mean and SD for the occurrence of each motion mode.

We define motion motifs as modes that occur in the real data much more often than in the shifted data. Because the distribution of mode occurrence between shifted datasets is close to Gaussian (not shown), we used a Z-score approach to define our threshold for defining when a mode is a motion motif. We assigned Z-scores to each mode defined by the difference between its mean prevalence in the real and shifted data, normalized by the standard deviation between shifted datasets. We used a threshold of *Z* > 3.1 which corresponds to *p* < 0.001 in Gaussian distributions, to address multiple hypothesis testing concerns over the 8 possible modes.

### Time Synchronization

To create a marker for synchronizing time between the cameras, participant gave a physical cue (high five) at the beginning of the session. We used Kinect Studio v2.0 to manually mark the cue in the time series, and to mark the beginning and end of the rounds.

### Space Calibration

We recorded a short excerpt prior or post recording sessions. In this excerpt, an experimenter posed statically in the middle of the room. The body, as seen simultaneously from both sources, was used to establish a common reference frame for the session’s recorded data.

## Results

### Two depth-sensing cameras measured the body coordinates of dyads in scientific conversations

Twelve pairs of scientists participated in the study. The pairs had frequent scientific conversations in the past. We asked the pairs to have three rounds of conversation in a room which is a typical setting for scientific discussions. The three rounds, each lasting 10-15min, were as follows: (i) Participant A shared a significant personal scientific experience. (ii) Participant B shared a significant personal scientific experience. (iii) Joint discussion about a question posed by the experimenter aiming to reach agreement about the answer. Dyads were asked to behave as they normally do in scientific conversations.

To measure body motion during the conversations, we employed two depth-imaging Kinect cameras [70] (Fig 2) with an additional non-depth video camera. We recorded the 3D positions of the skeletal joints of each participant (Fig 3).

### Torso motion provides a low-dimensional signal that captures most of motion variance of each individual

In order to define dyadic modes, we begin with defining individual modes. Then, we will combine the individual modes to produce dyadic modes. To define individual modes, we performed principal component analysis (PCA) on the speeds of the skeletal joints of each individual, normalized to zero mean and unit variance. The speed of a joint is defined as the root of the sum of its three squared velocity components. We analyzed 13 joints—head, neck, spine, shoulders, elbows, wrists, and pelvis. Leg coordinates showed noise due to tracking errors and were not included.

**Fig 5:**
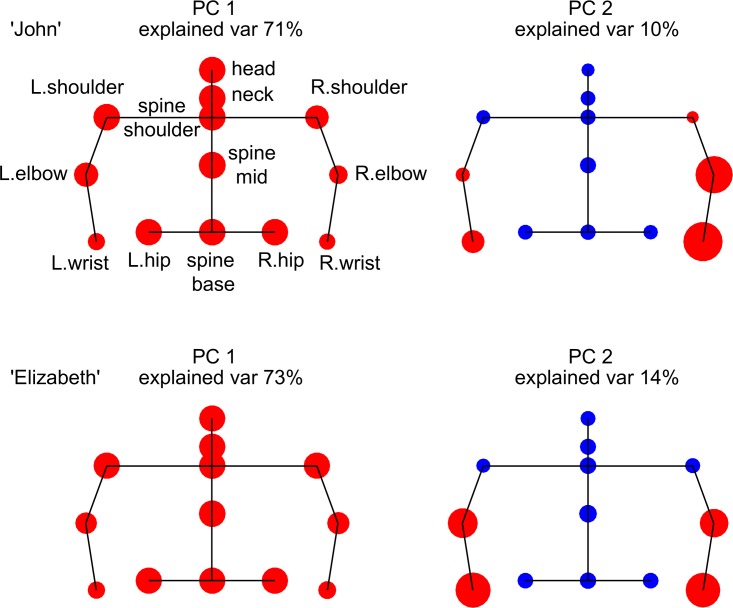
Principal components of the speed of 13 joints of individuals from different dyads. Circle size represents the magnitude of joints’ loadings in the PC. Color marks the sign of the loading: positive (red) or negative (blue). PC1 is similar across participants and corresponds to about equal loading in each upper body joint. PC2 is more variable between participants and shows loadings primarily on one or both arms.

We find that that the first principal component (PC1) explained most of the variance for each person (57%-95%). The first PC was similar between participants (*R*^2^ = 0.87 on average). PC1 describes rigid body motion of the torso, with about equal loadings for the shoulders, spine and pelvis (Fig 5).

The second PC (PC2) corresponded primarily to hand motion, and explained less of the variance (<10% on average). This component also varied in its loadings between participants: for example, some participants had PC2 that corresponded to symmetric motion of the two hands, whereas others had contributions mostly from one of the two hands (Fig 5, see SI for more details). Higher PCs were not significant.

Due to the predominance of PC1, which describes rigid torso motion, and its similarity between participants, for the remaining analysis we focused on the motion of the torso of conversants. As a proxy for rigid torso motion, we use the most robustly resolved body coordinates, the two skeletal coordinates at the sides of the pelvis (Fig 3). The pelvis coordinates showed high mean correlation with other torso joints (*R*^2^ = 0.84), and describe PC1 well because they have loading primarily on PC1, and not on higher order PCs.

**Fig 6:**
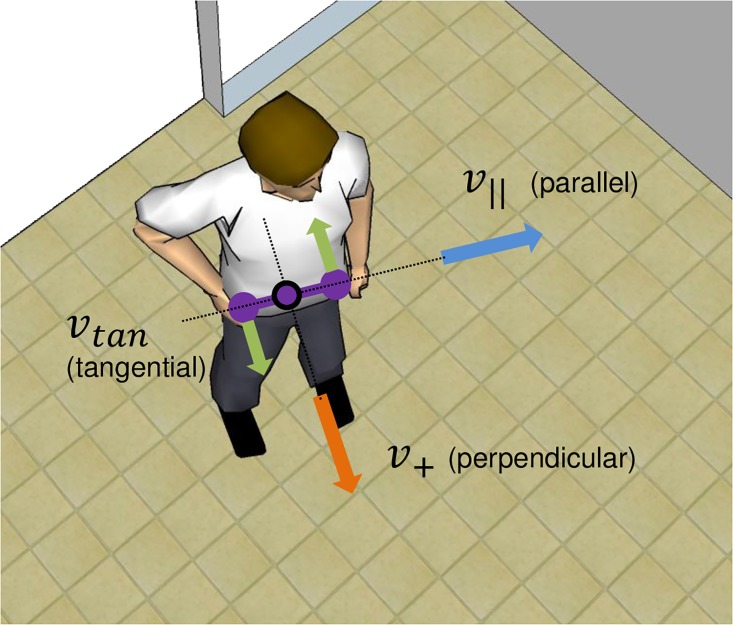
Illustration of the torso coordinate system. The complete 6-dimensional system consists of three velocity components for each pelvis in the horizontal plane for each of the two participants.

Further analysis shows that speed of the torso in the up-down direction is small relative to the speed in the horizontal direction (RMS speed ratio of 36%). It is thus sufficient as a first approximation to describe the torso motion in the horizontal plane. We therefore define a dyadic coordinate system based on the two torsos in the plane. This system has 6 coordinates, with three coordinates per person: *v*_||_ is velocity parallel to the person’s pelvis, *v*_+_ is velocity perpendicular to the pelvis, and *v*_*tang*_ is twist around the center of mass of the pelvis (Fig 6).

### Motion modes are defined by principal components of dyadic motion in a time window

We now turn to consider the motion of the dyad as a system, using the coordinates for the two torsos defined above. We find using cross-correlation analysis on our sample that the mean correlation time of the two persons’ center-of-mass speed is about 3sec. This supports a window-based approach in order to discover brief intermittent spatial modes of motion (cf. Condon and Sander [76] and Boker et al [71]), instead of attempting to analyze the full time series in one piece which would mix intervals of motion with different characteristics. We segmented the motion data into 2-sec time windows (similar results were found when using window sizes of 3 and 4 sec).

Some of the 2-sec segments corresponded to moments of stillness in which both persons had low velocity. We define these as still segments, according to a threshold on the RMS speed (Methods). The fraction of still segments varied between 7-78% between dyads, a range due in part to the large variation between participants in the propensity to be still (see below). Segments in which only one person moved (had RMS speed above threshold) were classified as solo segments, which accounted for 20-68% of the segments. In the remaining segments, which we call co-active segments, both persons moved.

In each 2-sec window, we computed the PCA of the 6-coordinate torso velocity data of the dyad. We retained the first PC in each window. The first PC accounted for 70% ± 13% (mean ± SD) of the variance in each window, and therefore captured the basic dyadic motion in each time segment (Fig 5). This PC is thus considered the dyadic *‘motion mode’* of that time window.

**Fig 7:**
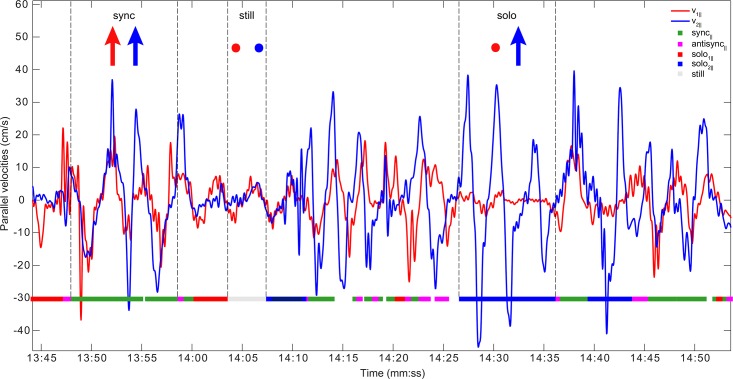
An example of pelvis parallel velocity profiles of two conversing participants from dyad 1. Player 1 (red) and player 2 (blue) parallel pelvis velocity is shown, after the smoothing described in Methods. Motion modes for each moving 2-sec window were classified into pure modes, and color-coded on the horizontal colored bar. Examples of periods of consecutive parallel-sync, mutual stillness and solo motion are indicated (dashed lines and arrows).

We classified each motion mode according to its parallel and perpendicular components (loadings of the PC, see Methods). We neglected the tangential velocity due to its small contribution to the overall speed (RMS ratio of parallel, perpendicular, and tangential components is given by 1:0.7:0.2 respectively). Fig 1 shows the eight possible pure modes in which each person does parallel, perpendicular or no motion. An example of the velocity traces of a dyad and the modes in several selected intervals is shown in Fig 7. We find that the most common co-active modes are parallel-synchronized torso motion, and the most common solo mode is parallel torso motion by one person while the other is still.

### Parallel-synchronized torso motion and mutual stillness are motion motifs

A central part of our approach is to determine which part of the motion results from the dyadic interaction, rather than from the motion characteristics of each of the two individuals. We therefore asked which motion modes occur more often than expected by chance given the motion statistics of each individual. We term such modes *‘motion motifs’*, borrowing the term from biology, in which sequence motifs and network motifs are commonly used terms for over-represented features relative to shuffled controls [77, 78].

To detect motion motifs, we prepared a control dataset in which the short-term correlations in the motion were broken, but the statistics of each person were maintained (cf. pseudo interaction clips by Bernieri et al. [31]). The controls were generated by cyclically shifting the time axis of one of the two participants by at least one minute. We repeated the motion-mode analysis on M = 30 shifted datasets to obtain a mean and SD for the occurrence of each motion mode.

We define motion motifs as modes that occur in the real data much more often than in the shifted data (we used a threshold of *p* < 0.001 to address multiple hypothesis testing concerns over the 8 possible modes).

Notably, our shifted controls considered the time series of the two participants within a dyad. This differs from another common method to produce baselines, in which participants’ non-shuffled time series are paired with the time series of another participant (i.e., one with whom they did not actually interact) to form a “virtual pair” [79–81]. The problem with baselines generated from non-interacting pairs is the heterogeneity of the individual’s motion, e.g. some individuals are still for 10% of the time, others for 80% of the time. Thus, baselines from non-interacting dyads introduce a large bias because of this heterogeneity (data not shown). Shifted baselines from interacting dyads preserve the statistics of each person in the dyad and are thus a more stringent control. We also refrained from baselines involving shuffled time series to preserve the spectral structure of the signal [82, 83].

Another way to think about motion motifs is to consider the null hypothesis that dyadic modes are simply the result of chance. Hence the probability of a dyadic mode AB made of individual modes A and B is the product of the probability that individual one shows mode A and individual two shows mode B. A stringent control for this is to shift the dynamics cyclically, thus breaking correlations but preserving each persons statistics of their individual modes. Motion motifs are dyadic modes for which one can rule out the null hypothesis according to a predefined statistical criterion.

**Fig 8:**
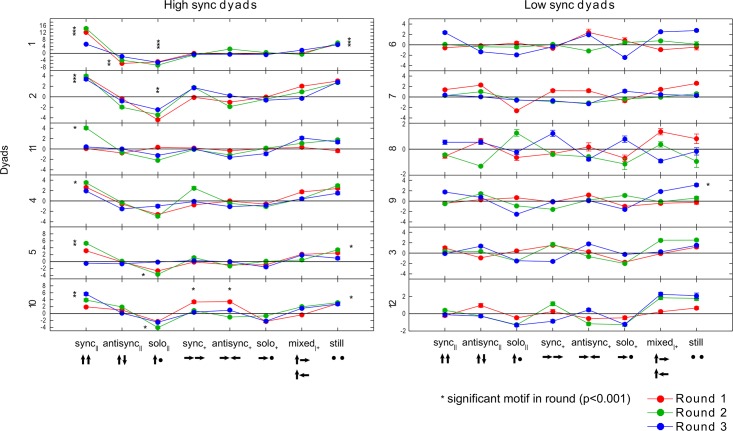
Two motion motifs recur across many of the dyads. Z-scores of the eight dyadic torso motion modes for all dyads across rounds 1-3. Z-scores are the number of standard deviations that the motion mode occurs in the real data compared to cyclically shifted control data. Asterisks indicate Z-score > 3.1, which corresponds to *p* < 0.001 (the data distribution is close enough to Gaussian to warrant a relationship between Z-score and p-value). Two modes are motion motifs in many of the dyads: parallel synchronized torso motion, and mutual stillness. Solo parallel torso motion is an anti-motif, with large negative Z-scores in many dyads. Half of the dyads showed no significant motifs (right panel). Error bars by bootstrapping are smaller than the marker size.

In our sample, we find that only two of the eight possible modes qualify as motion motifs (see Methods). Half of the dyads (6/12) showed parallel torso motion in synchrony as the main motif (Fig 8, *p* < 0.001). Still segments were also a significant motif in 4/12 dyads (*p* < 0.001). About half of the dyads did not show any significant motion motifs. Taking all data together, the parallel-synchronized and still modes are the most different from the shifted control, and other modes are closer to the shifted controls. The data also showed anti-motifs: modes which occurred less often than in shifted controls. The primary anti-motif was solo parallel motion (4/12 dyads, *p* < 0.001). We conclude that about half of the dyads showed motion motifs, and that the same motifs and anti-motif recur in different dyads.

### Dyads show individuality in their mode distributions

**Fig 9:**
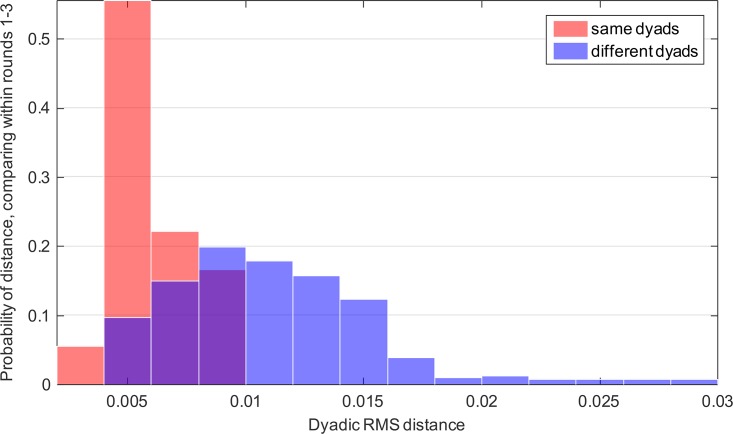
Motion mode distributions of a dyad are more similar to itself in other rounds than to other dyads. Euclidean distance between the motion mode probability distribution functions is shown, intra-dyad (red) and inter-dyad (blue). The two histograms are significantly different according to the two-sample Kolmogorov-Smirnov test (*D* = 0.6, *p* < 10^−6^).

Finally we demonstrate that the present approach can be used to ask about the characteristic signature of the motion of a dyad, which can be termed dyad individuality. We asked whether dyads in our sample show individuality in their use of motion modes. For this purpose we compared the probability distributions of motion modes between dyads (Methods). We find that the mode distribution of a dyad is more similar to itself in different rounds than it is to other dyads (Fig 9), *D* = 0.6, *p* < 10^−6^, two-sample Kolmogorov-Smirnov test). This means that regardless of the task—that is, regardless of which person presents the scientific topic, or the joint task—the distribution of motion modes are characteristic of a dyad.

To test whether this individuality lasts for longer times, we performed an additional round of experiments with 5 of the dyads 6 months after the first experiments. The dyads were given a task similar to round 3 of the first experiment. We find that individuality is maintained over this 6 month interval: a dyad is significantly more similar to itself six months ago than it is to other dyads (*D* = 0.4, *p* = 0.01, two-sample Kolmogorov-Smirnov test).

In addition to constancy of dyad characteristics over time, we asked about the constancy of individual participants’ characteristics. We studied individuals’ PC2 (Fig 5), which primarily represents hand motion. We compared PC2 for the individuals that did the experiment twice, with a 6 month delay. Six out of ten participants who participated twice had a significant PC2. For these participants, PC2 was significant in both the primary session and the delayed one. We find that PC2 is more correlated over time for the same participant (*R*^2^ = 0.98) relative to correlation of PC2 across participants (*R*^2^ = 0.75). In addition, we find that the fraction of time that a person is still is correlated over the 6-month period (*R*^2^ = 0.94).

## Discussion

We presented a method to find dyadic modes of motion in naturalistic joint action. The method involves three steps: defining individual modes, defining dyadic modes made of combinations of these individual modes, and defining motion motifs which are dyadic modes that occur more often than chance. We demonstrated this on a sample of scientific and non-scientific conversations.

Understanding human motion in conversations is challenging due to the complex high-dimensional signal obtained from measuring many body coordinates. Here we presented an approach to simplify this motion and find salient modes. This is based on a series of steps which reduce the data, keeping only the main elements [12]. We first focused on a single person in the dyad, finding that most of the individual motion variance is due to rigid-body torso motion. We therefore used the two torsos as the minimal unit when we explored dyads. Dyads can show at least eight modes in which the two torsos move in parallel, perpendicular, etc. We analyzed the two-torso dynamics in 2-sec temporal windows, a timescale over which the two person velocities are typically correlated. We defined motion modes in each window as the first PC of the dyadic torso motion: a succinct description of the dyadic motion in each 2-sec segment. Finally, we introduce the notion of a motion motif as a motion mode that occurs more often in the real conversation than expected based on the two persons’ individual motion statistics.

We measured conversations of 12 dyads of scientists as a proof-of-concept. The small sample size warrants caution in generalizing the specific results. In our sample, dyads showed only two dyadic motion motifs out of eight possible modes, and these motifs recur across dyads. The recurring motifs are parallel sync torso motion and still segments. Dyads showed individuality of motion mode use across tasks. This individuality is maintained across time (six months) in a subset of 5 randomly selected dyads for which a longitudinal analysis was performed.

The main motion motif in our sample, parallel-synchronized torso motion, corresponds to the two persons swaying from side to side in synchrony. Visual inspection of the videos shows that such sway occurs in each individual naturally as a result of shifting weight from foot to foot. We hypothesize that naturally occurring torso sway provides a good signal for entrainment between the two conversing people. This mutual entrainment can enhance rapport [31, 32, 62, 72, 84]. In a sense, torso sway is like a pendulum and two people synchronize just like two coupled pendulum clocks would. Unlike clocks, however, people tend to exit synchrony as well [85, 86]. The periods of parallel torso synchrony in our sample averaged 2.9 sec. A second main motion motif in this sample was mutual stillness, which can also be considered a form of synchronized behavior (being still together, [87]).

The prevalence of parallel torso motion in our sample differs from a previous report of perpendicular pelvis motion as a main component in a dyadic task [68]. This difference might stem from the setting of the experiments. In the previous study, participants were asked not to move their feet for technical reasons related to the measurement. In the present case, participants moved their feet naturally. Perpendicular motion occurs in our experiments, but synchrony specifically occurs most often in the parallel direction.

The present method has several limitations. First, participants need to mind the cameras and refrain from self or mutual occlusions and avoid leaving the frame. This limits the naturalistic aspects of the behavior. Second, the position and target direction of the cameras must be carefully adjusted. Kinect depth sensing cameras project IR beams that interfere between the two cameras and are reflected by obstacles. Experimenters need to consider reflecting obstacles such as windows and mirrors and minimize the mutual projection between cameras. Third, the current approach was designed to study the motion of dyads. In principle, the same analysis steps may prove fruitful in group interactions as well, provided that depth motion data can be reliably collected from groups.

This study used a small overall sample size (n = 12), and an even smaller sample size in the longitudinal analysis (n = 5). It used non-naive subjects who were familiar to each other and were acquainted with the matters of their discussions [88]. Additionally, the subjects and the experimenter were aware of the study focus on body language, which might lead to stronger effects than when studies are run blindly [89]. This study was done in a single setting and culture, and future work can test its generality by examining conversations in other settings and cultures. Given that synchrony patterns change as a result of conversation goals and context [11, 21, 52, 66], we believe that the movement patterns identified in this sample may be characteristic of the current context (including the movement restrictions in the study instructions). It would therefore be important to explore motion motifs in other contexts. There is more to explore with the higher-order PCs of motion of individuals, especially hand and arm motion (SI) [90]. Moreover, it would be of interest to work in a coordinate-independent framework in order to avoid potential caveats, as suggested by Sternad et al [91].

Future work can also study how the basic motion modes found here correlate with the content level of the conversations [40, 46, 49, 53, 92–95]. Our approach provides a framework to ask how body motion correlates with properties such as speech turns [48, 58], mutual understanding and misunderstanding, confusion and frustration, rapport [31, 32, 62, 72, 84], introduction of new ideas, and creativity [70]. For this purpose, we made all software and data publicly available.

In summary, we present an approach to define dyadic modes and motion motifs from complex body motion data in joint action. We define motion motifs as dyadic modes that occur more often than expected from the single-person motion statistics. We demonstrate this on a sample of scientific conversations. The present approach may be applied more generally to understand the embodied aspects of joint action.

## Supporting Information

S1 Fig Individual PCA, entire dataset. We present the significant PCs based on the PCA of speeds of 13 joints for all participants. When combining all individual data, PC1 is the only significant PC representing body rigid motion.
(PDF)

S2 Fig Torso parallel motion modes distributions, entire dataset. Torso parallel motion modes distribution of all dyads across rounds 1-3. Pure modes correspond to specific regions in histograms. Dyads exhibit individuality: they appear more similar to themselves across rounds than to other dyads.
(PDF)

S3 Fig Distribution of pure modes in dataset. Distribution of pure modes in real data compared to shifted datasets in the entire dataset. Error bars by bootstrapping are smaller than the marker size.
(PDF)

S4 Fig Participant displacement dataset. We plot the projection of the left and right hip joints’ positions on the horizontal plane. Each plot shows data for both participants in a specific round: participant 1 left (red) and right (green) hips, and participant 2 left (cyan) and right (purple) hips. To better visualize we down-sampled the data by 300. These plots show different spatial occupation and displacement patterns exhibited by different participants. Moreover, this data suggest a participant-characteristic formations of position.
(PDF)

S5 Fig Torso displacement dyadic motion modes by PCA. In the main text, we analyzed velocities (temporal derivative of position). Here we analyze positions. We perform PCA on the left and right hip joints’ positions of both participants on the horizontal plane. The significant PCs are visualized as an arrow of length equal to one SD of the data along that PC. The red (participant 1) and blue (participant 2) arrows illustrate the coordinated motion pattern: coordinated direction and amount of relative displacement associated with a particular PC. Note that PCs are determined up to a sign. The significant PCs are determined by their explained variance (percentage shown) relative to shuffled controls where dyadic correlations in displacement were broken. Accordingly, dyads differ in their amount of significant PCs. These plots support the finding that synchronized parallel (sideways) motion is a motion motif.
(PDF)

S1 Text Full instructions for participants. (DOCX)

S2 Text Best practices. (DOCX)
