# Supplementary figures and images for: A reduced-dimensionality approach to uncovering dyadic modes of body motion in conversations
Source: PLoS One. 2017 Jan 31;12(1):e0170786. doi: 10.1371/journal.pone.0170786 (PMC5283650; doi:10.1371/journal.pone.0170786)

Dvad 1

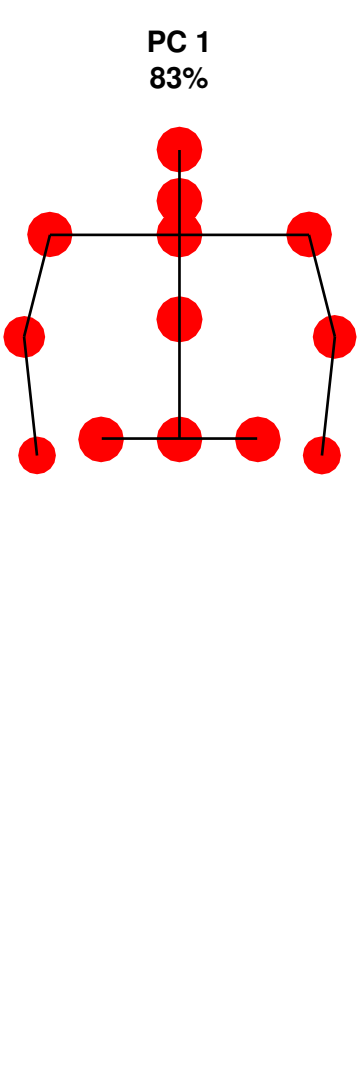

Dyad 2

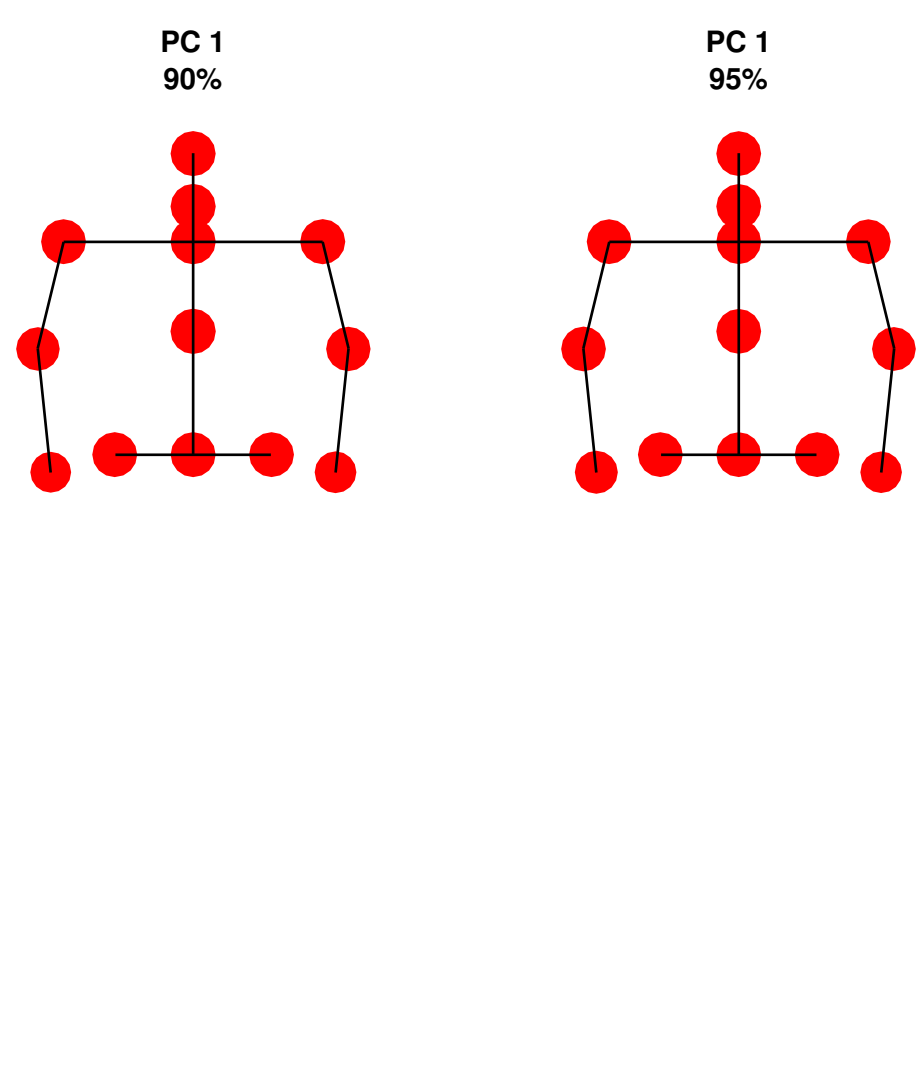

Dyad 3

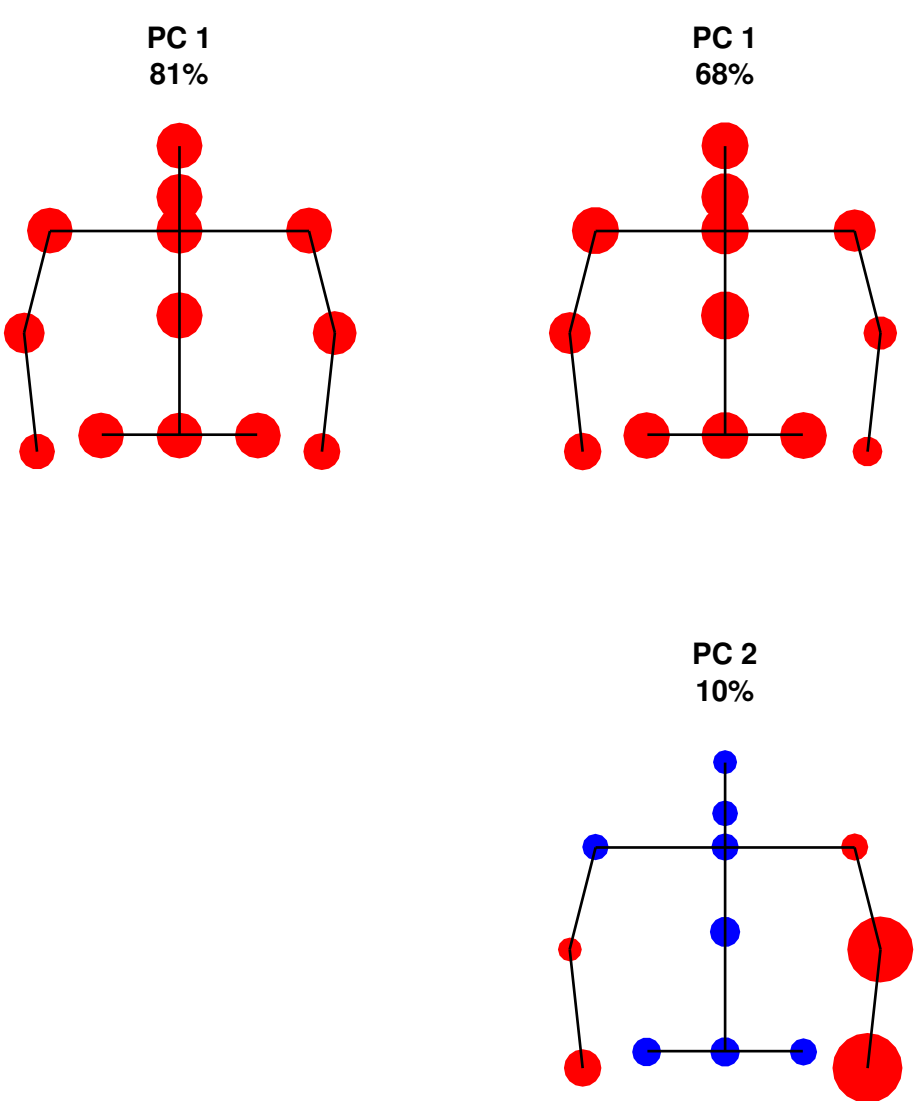

Dyad 4

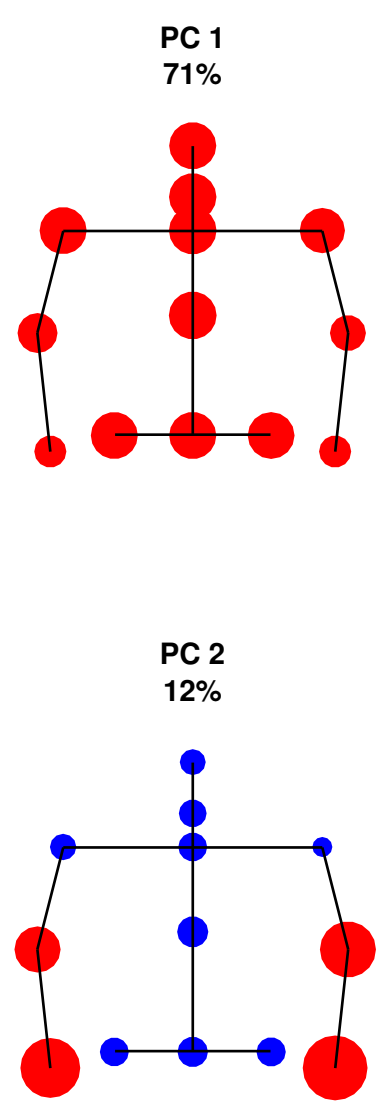

Dyad 5

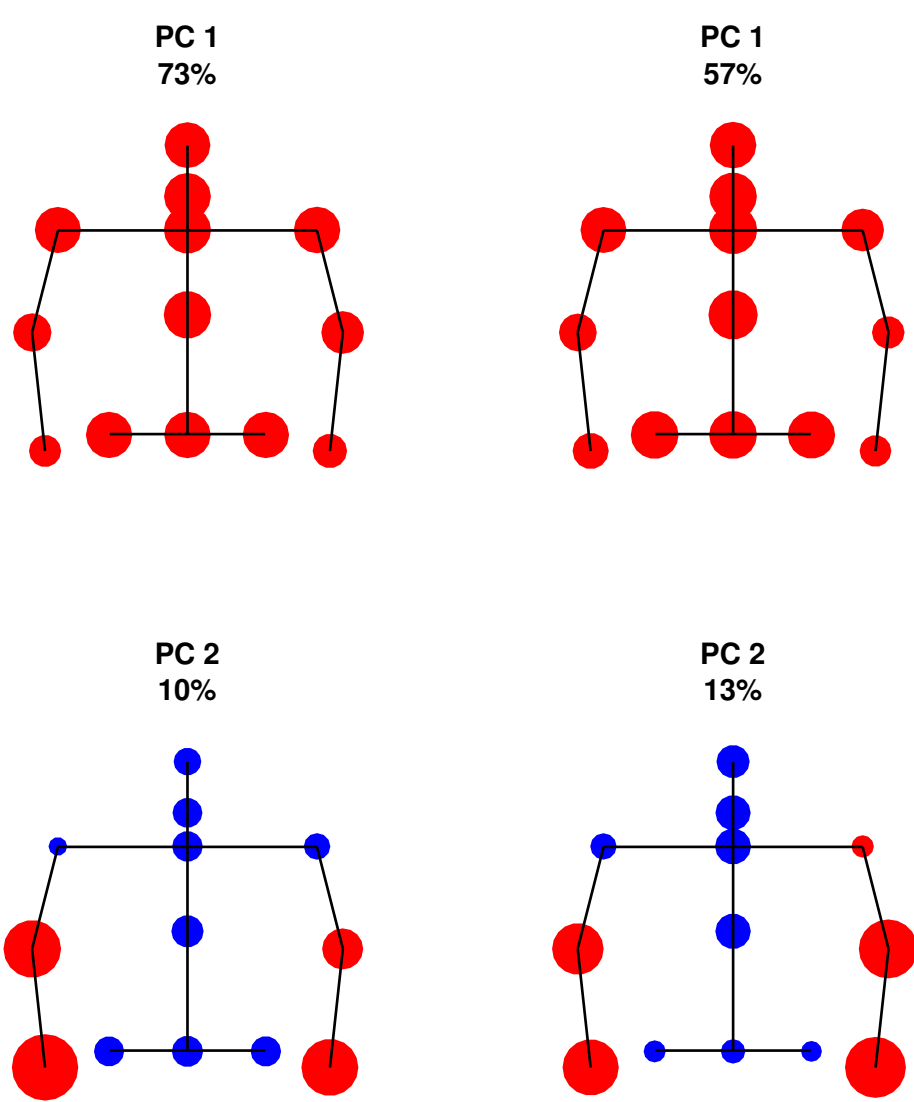

Dyad 6

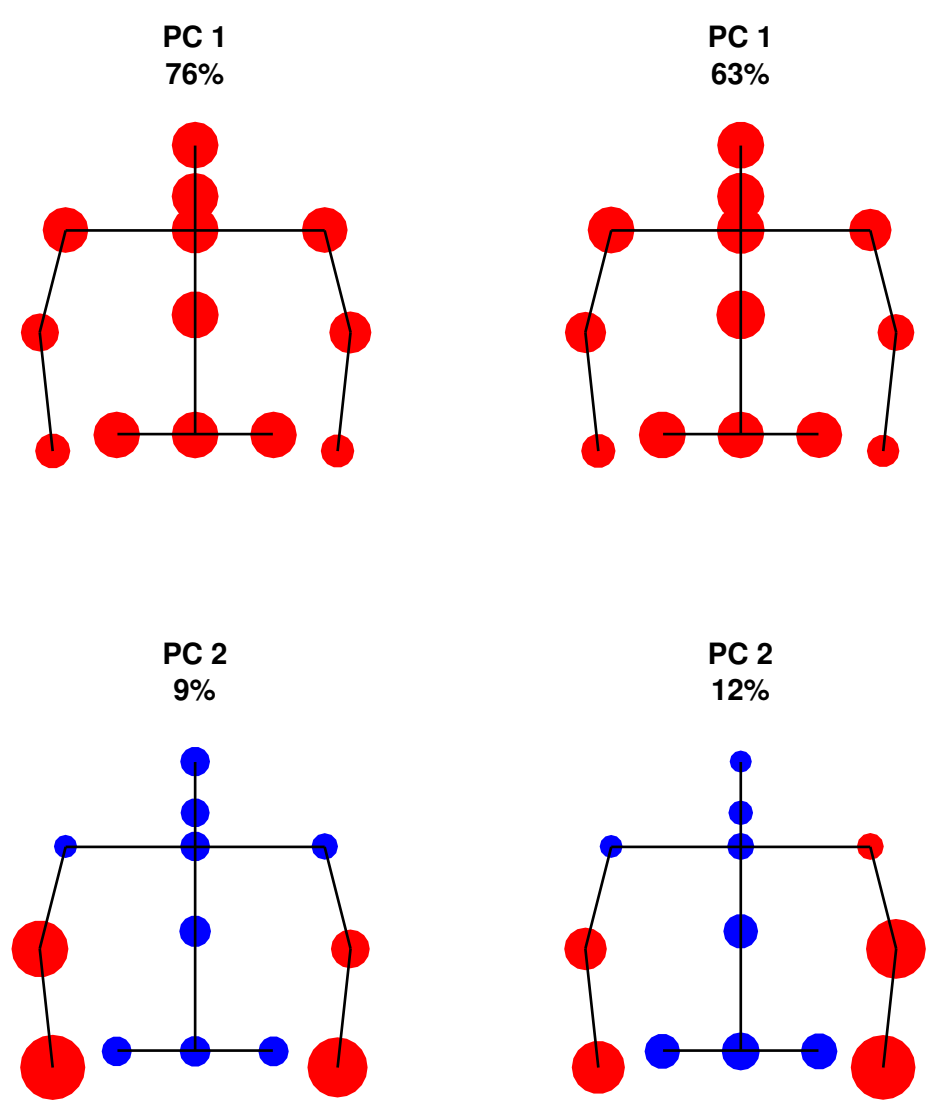

Dyad 7

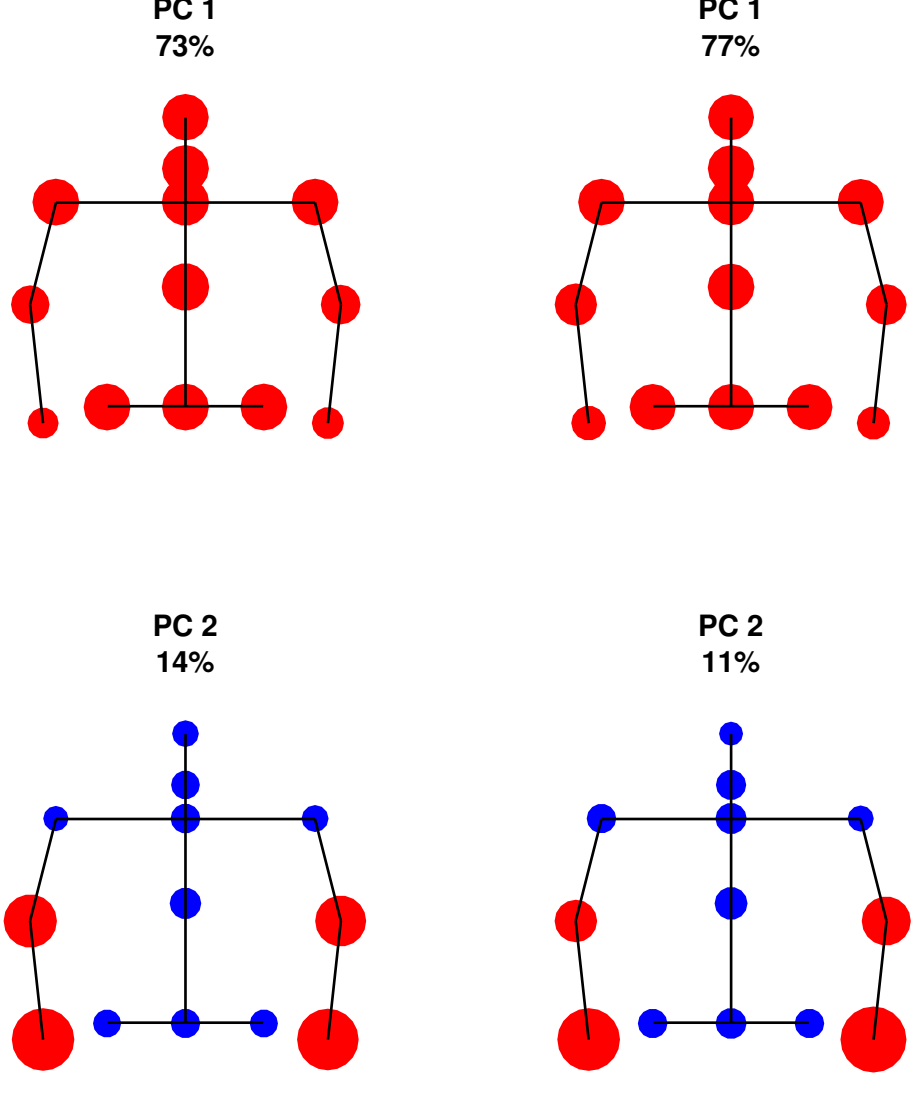

Dyad 8

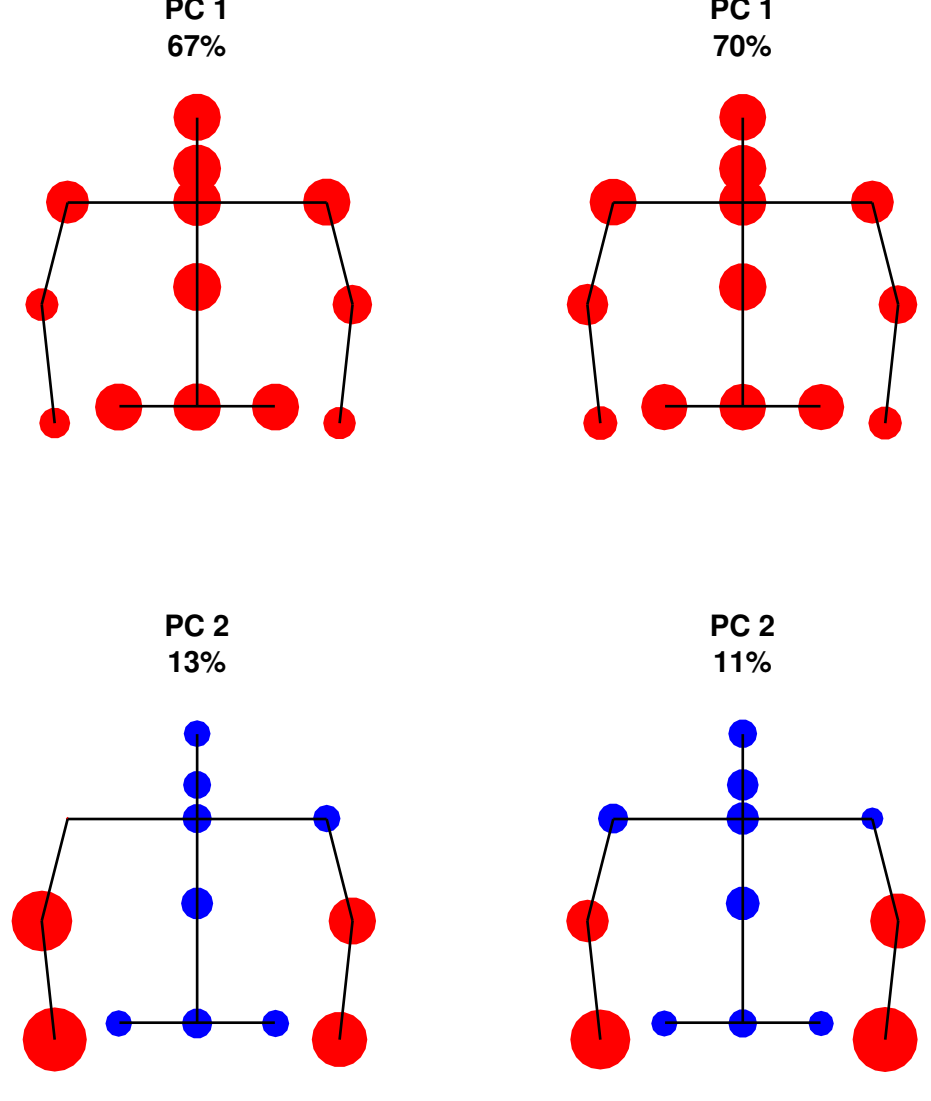

Dyad 9

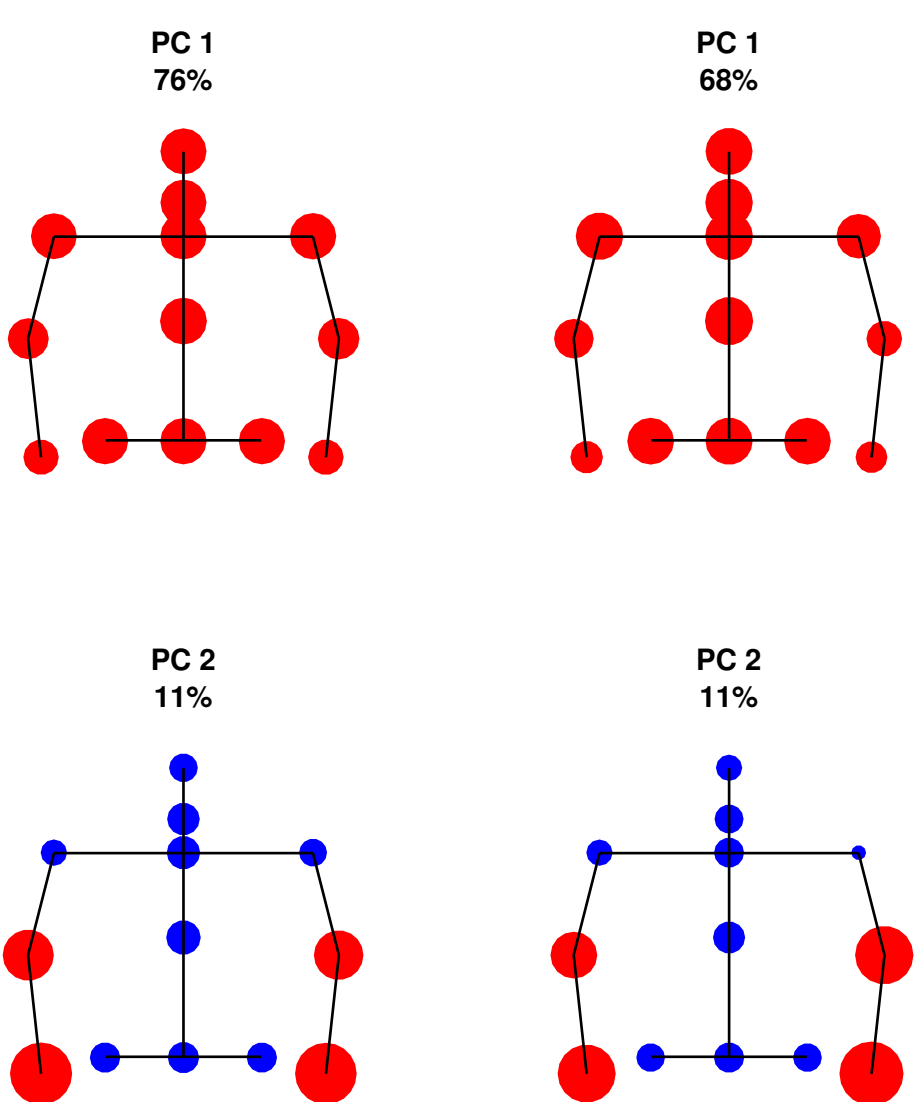

Dyad 10

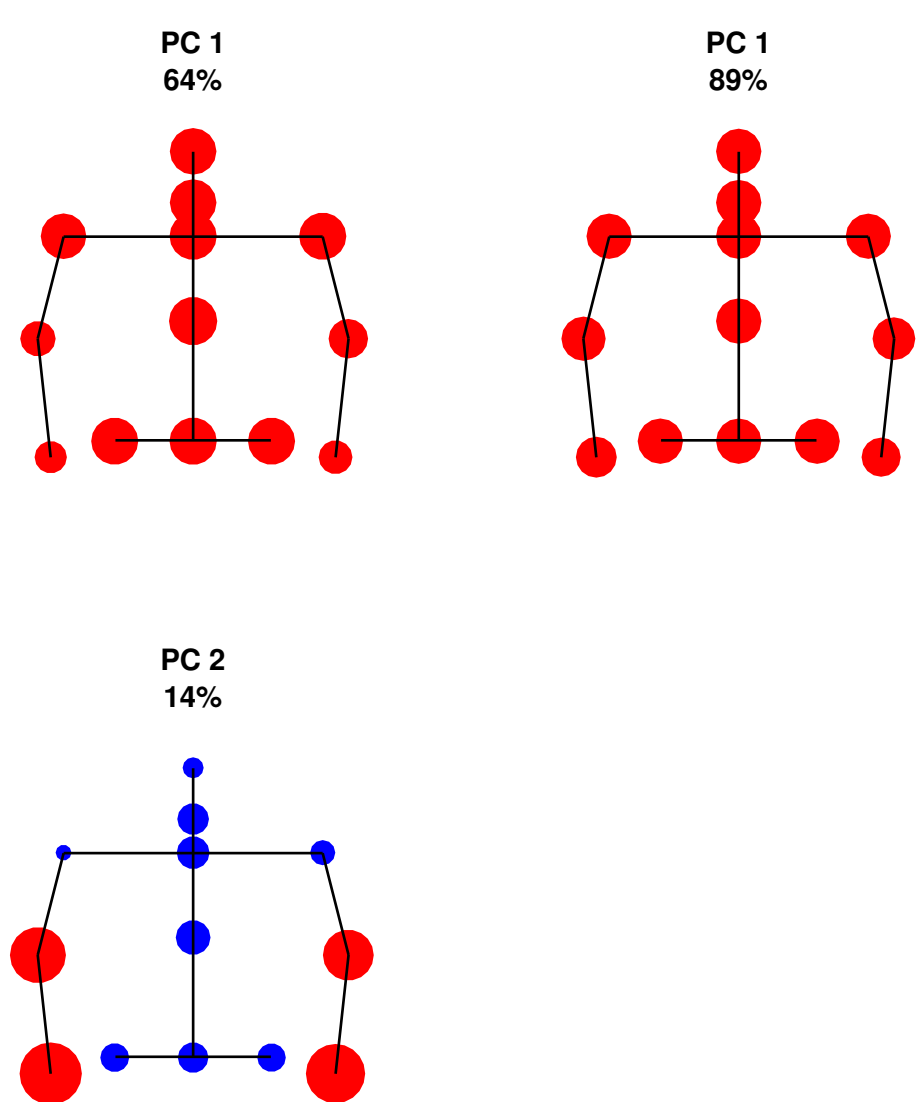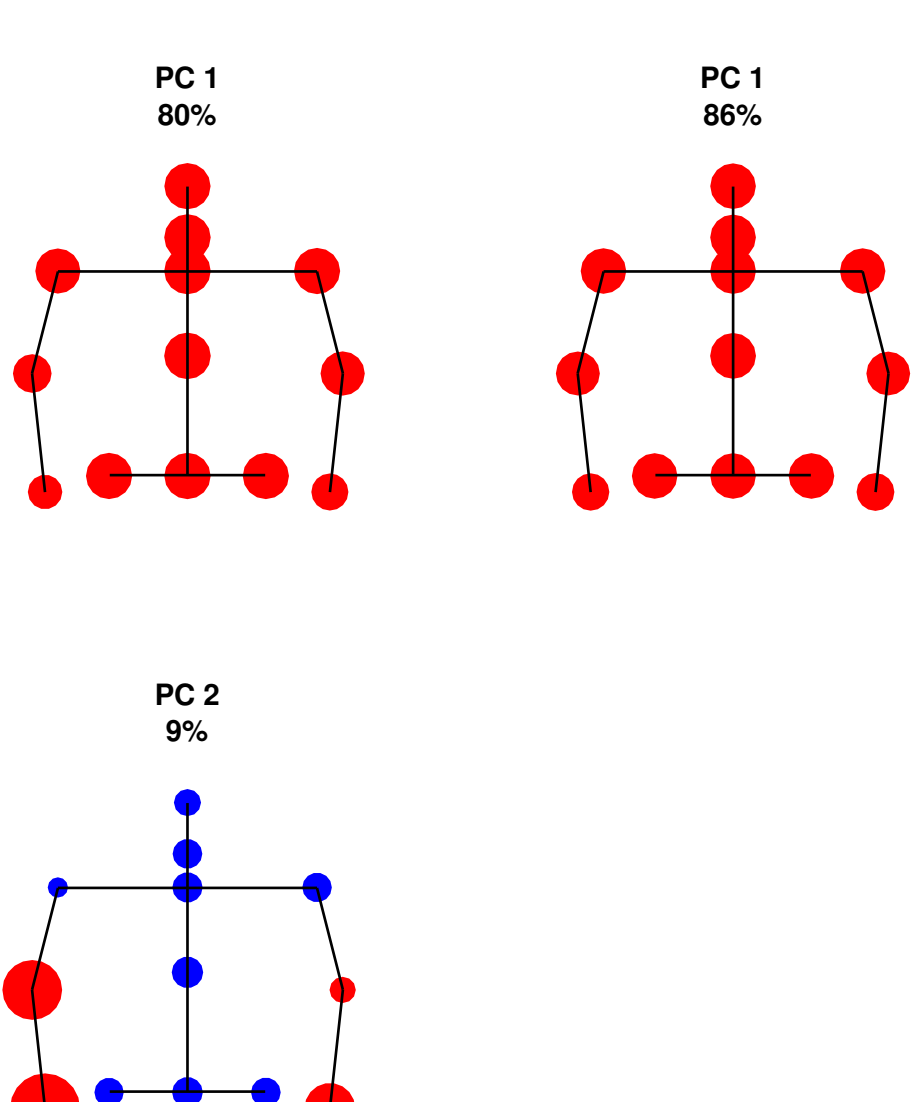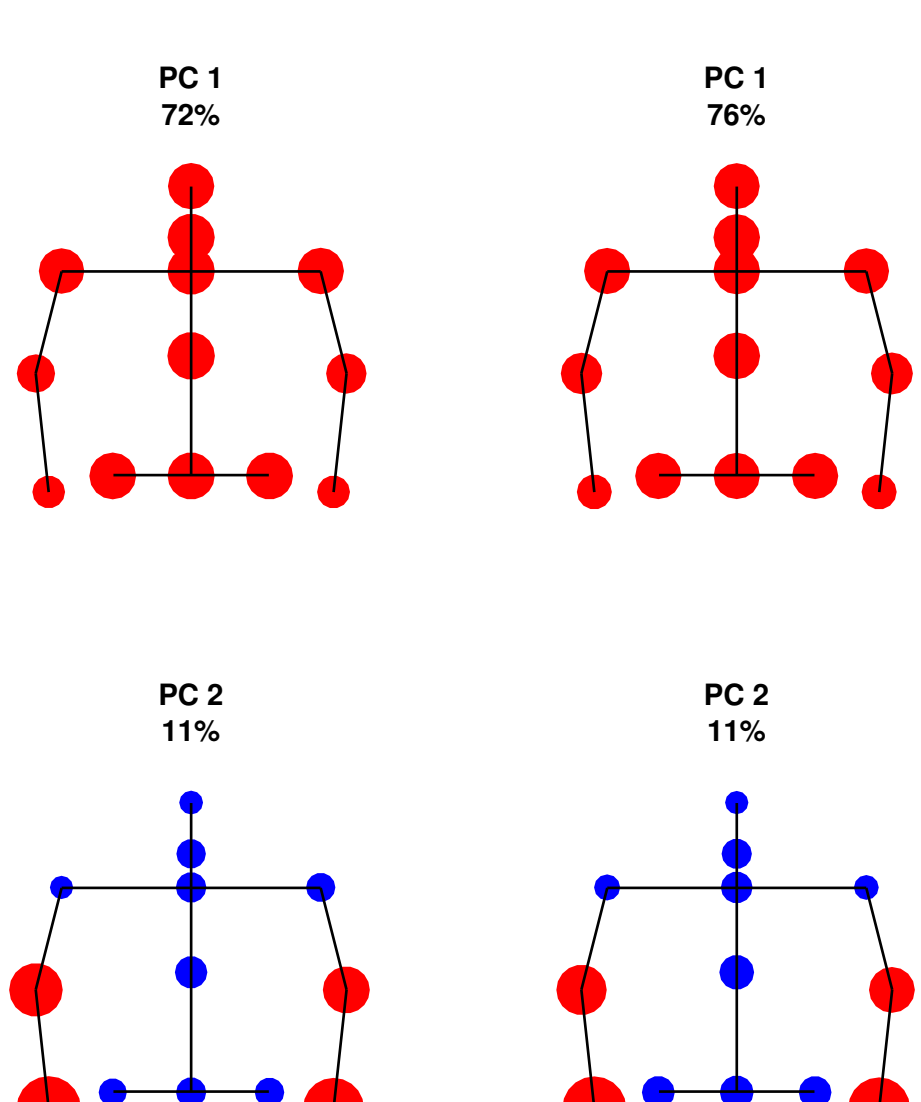

Supplement: S1 Fig — We present the significant PCs based on the PCA of speeds of 13 joints for all participants. When combining all individual data, PC1 is the only significant PC representing body rigid motion. (PDF) [file pone.0170786.s001.pdf]

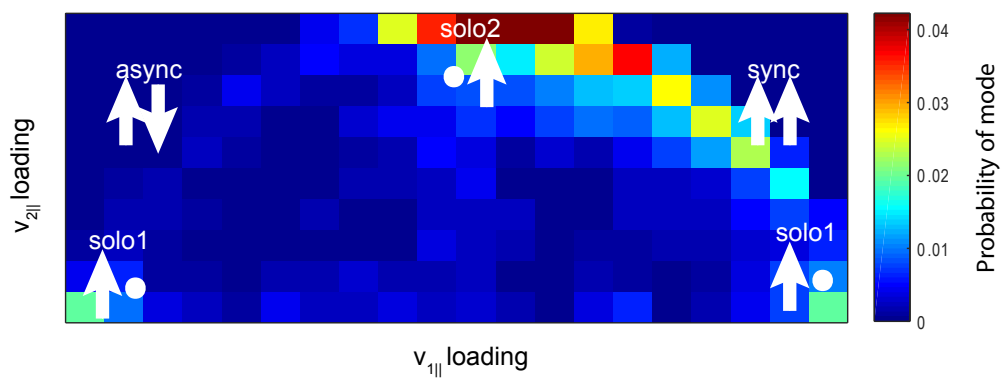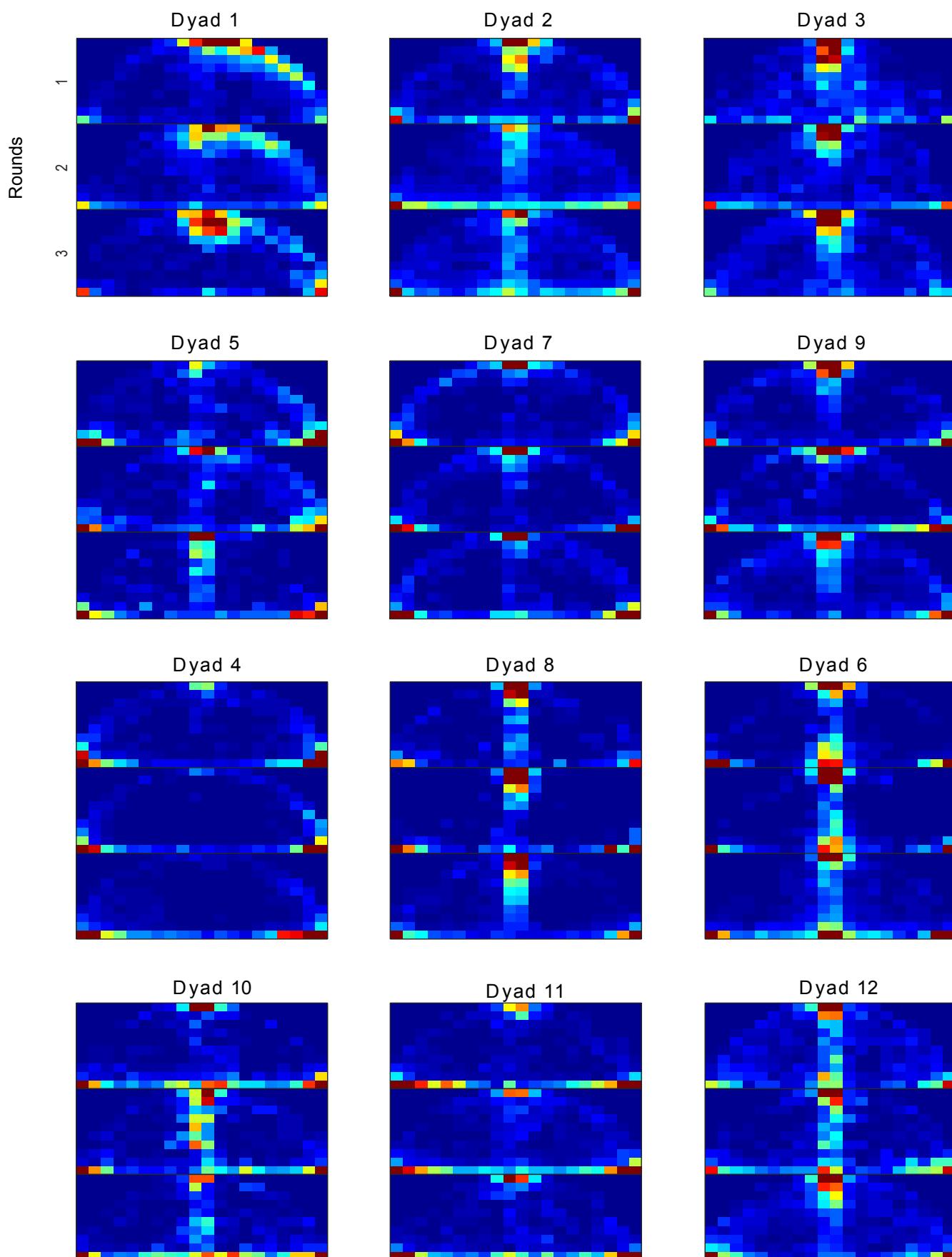

Supplement: S2 Fig — Torso parallel motion modes distribution of all dyads across rounds 1-3. Pure modes correspond to specific regions in histograms. Dyads exhibit individuality: they appear more similar to themselves across rounds than to other dyads. (PDF) [file pone.0170786.s002.pdf]

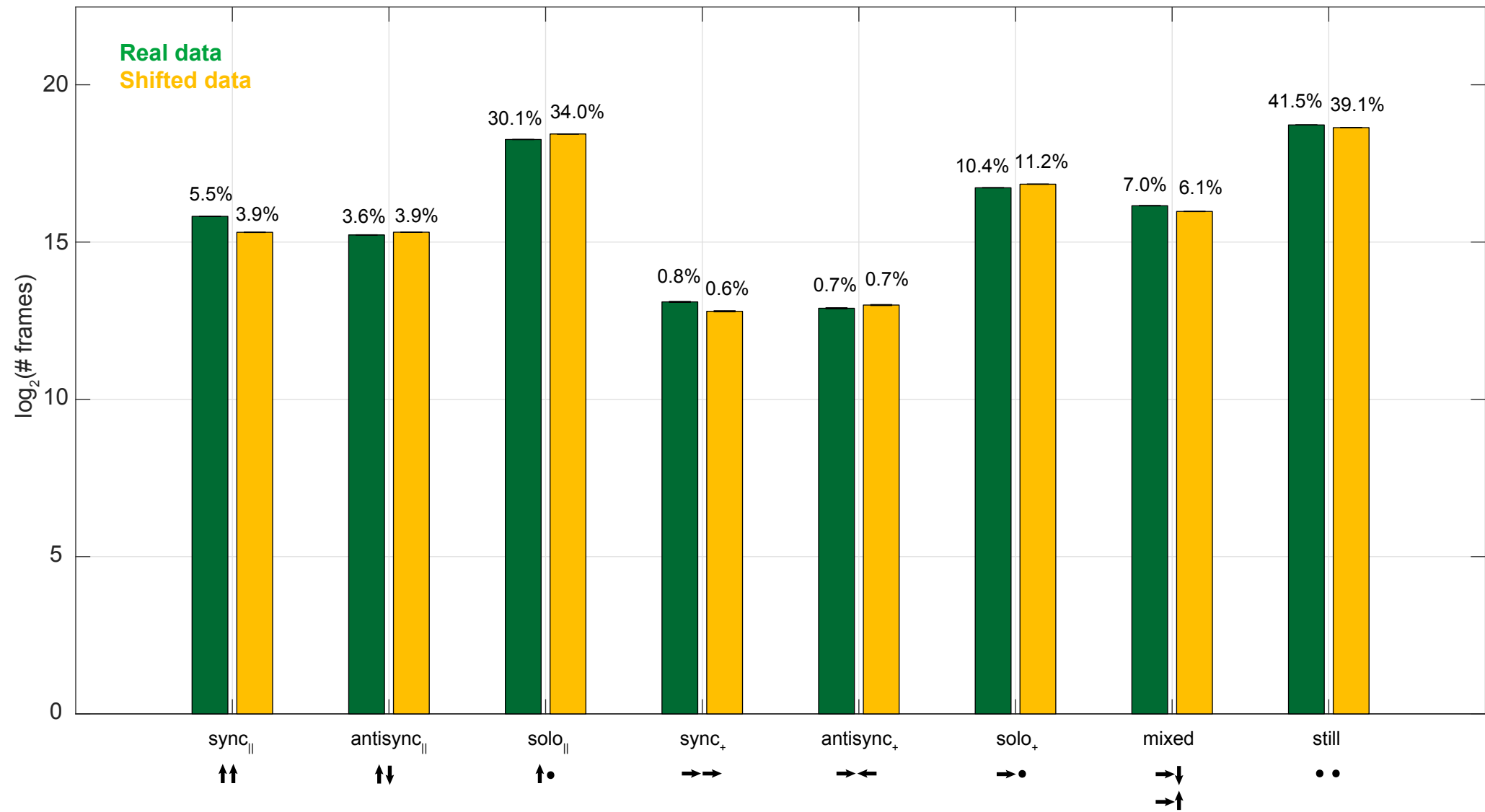

Supplement: S3 Fig — Distribution of pure modes in real data compared to shifted datasets in the entire dataset. Error bars by bootstrapping are smaller than the marker size. (PDF) [file pone.0170786.s003.pdf]

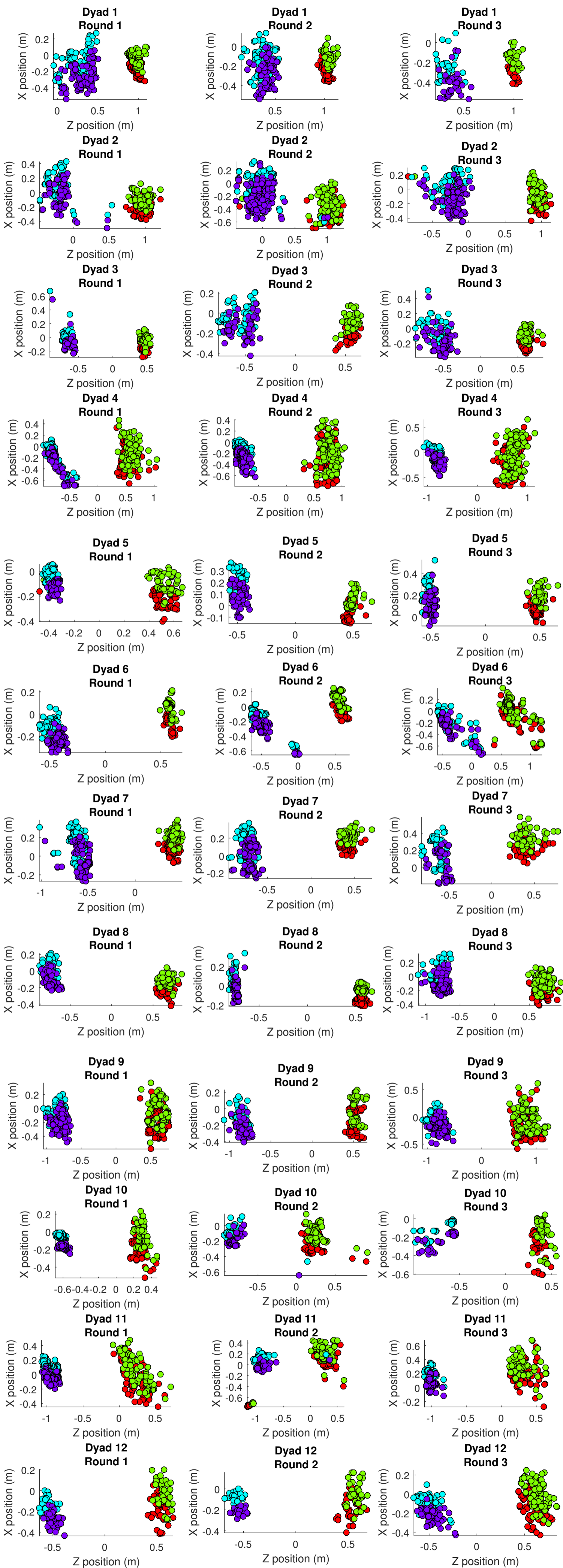

Supplement: S4 Fig — We plot the projection of the left and right hip joints’ positions on the horizontal plane. Each plot shows data for both participants in a specific round: participant 1 left (red) and right (green) hips, and participant 2 left (cyan) and right (purple) hips. To better visualize we down-sampled the data by 300. These plots show different spatial occupation and displacement patterns exhibited by different participants. Moreover, this data suggest a participant-characteristic formations of position. (PDF) [file pone.0170786.s004.pdf]

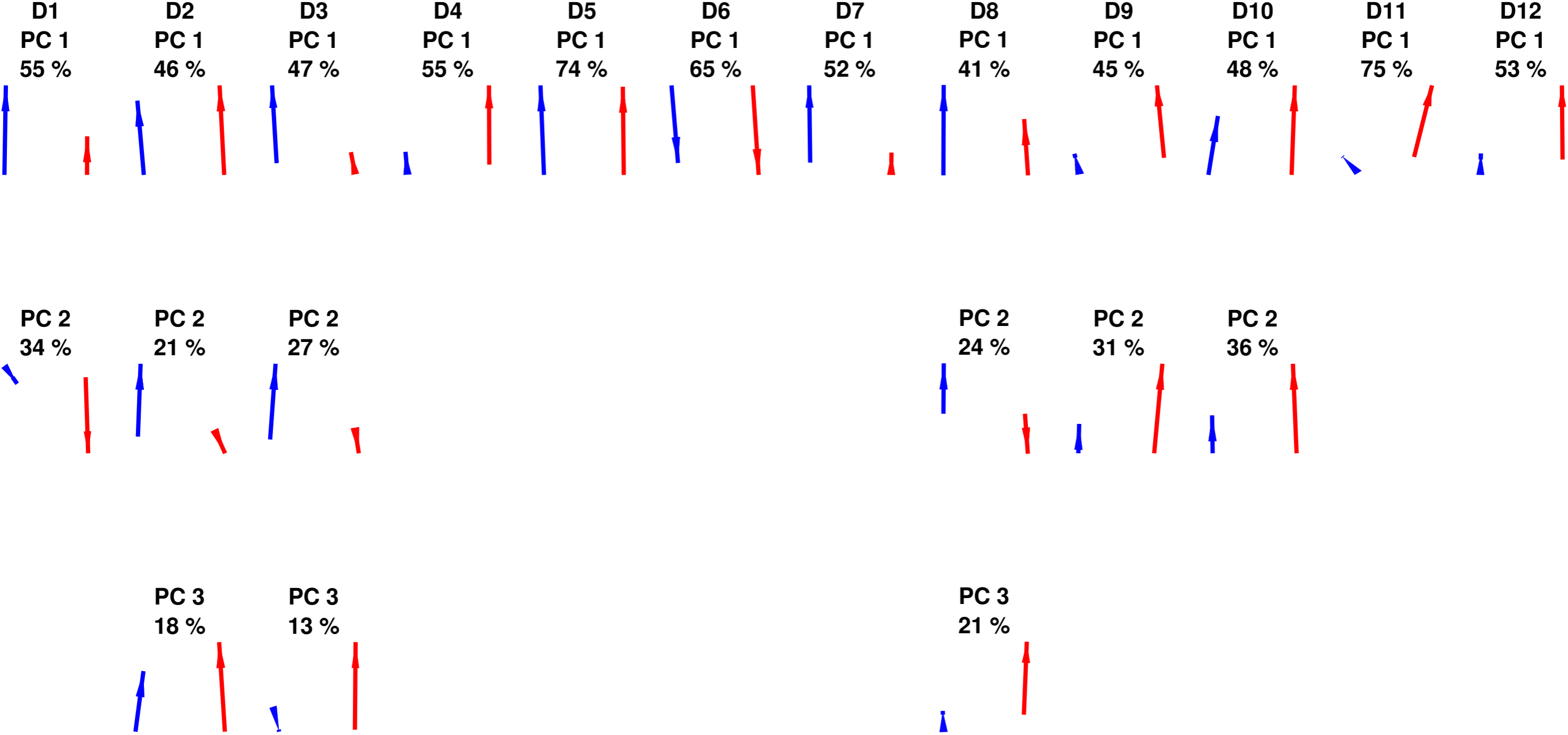

Supplement: S5 Fig — In the main text, we analyzed velocities (temporal derivative of position). Here we analyze positions. We perform PCA on the left and right hip joints’ positions of both participants on the horizontal plane. The significant PCs are visualized as an arrow of length equal to one SD of the data along that PC. The red (participant 1) and blue (participant 2) arrows illustrate the coordinated motion pattern: coordinated direction and amount of relative displacement associated with a particular PC. Note that PCs are determined up to a sign. The significant PCs are determined by their explained variance (percentage shown) relative to shuffled controls where dyadic correlations in displacement were broken. Accordingly, dyads differ in their amount of significant PCs. These plots support the finding that synchronized parallel (sideways) motion is a motion motif. (PDF) [file pone.0170786.s005.pdf]
