# Supplementary material for: A reduced-dimensionality approach to uncovering dyadic modes of body motion in conversations
Source: PLoS One. 2017 Jan 31;12(1):e0170786. doi: 10.1371/journal.pone.0170786 (PMC5283650; doi:10.1371/journal.pone.0170786)
Supplement: S1 Text — (DOCX) [file pone.0170786.s006.docx]

**Full instructions for participants**

Thank you for taking part in this study. In this experiment we will record 3 rounds of dialogues conducted between you. In each of the first two rounds, one of you is asked to consult the partner about a scientific experience or to share a significant moment of his/her scientific work. This may include any aspect, subjective or objective, of the experience. Most important is that the conversation is kept as natural as possible. In the final round you will be given a shared task in the form of a joint discussion where you will be expected to yield an outcome upon which you agree.

The length of each round is determined by your own natural interaction but shouldn’t exceed 15 minutes. You are free to spontaneously transition between round 1 and 2 and to choose who leads first. Once round 2 is finished you are required to let us know, and we will provide the last matter of discussion for round 3.

In order to have good quality of recordings we need to abide to certain rules:

1. Keep standing as if you were talking on a break in a conference.
2. The room is rectangular – Choose a side of the room and keep on facing each other without switching your side of the room.
3. Try to have the center point of the room in the middle point of your inter-distance.
4. Don’t use the whiteboard since this involves turning your back on the cameras.

This session is recorded. If you wish to, you can request to eliminate the data of you post-hoc.

Are there any questions? Please keep your phones silent and take a moment to refresh outside the room. I will call you in a few moments when we are ready to start.

[ Run Kinect cameras from both computers. Hide screens ]

[ Let subjects in the room ]

[ Time Sync: Instruct subjects to perform a high-five ]

Great so let’s begin. I’ll be waiting in my office once you are done with rounds 1 and 2. Call me then so that I’ll give you the joint matter for discussion. Enjoy!

[ Leave the room (close the door), waiting for the subjects to call for round 3 ]

[ rounds 1+2 ]

[ participants call the experimenter ]

Your joint matter for discussion is either:

1. What do you think will be the most important scientific or technological breakthroughs in the next 20 years? List and rate 3 of them.
2. Discuss and rate the 3 most important things that you think pupils should be taught at school.

What do you choose? [ response ]. Enjoy!

[ round 3 ]

[ Stop Kinect recording. Stop control camera ]

Thank subjects for participation, and notify them that they may be contacted later for subjective evaluation of the conversation. Answer questions.
