# Supplementary material for: A reduced-dimensionality approach to uncovering dyadic modes of body motion in conversations
Source: PLoS One. 2017 Jan 31;12(1):e0170786. doi: 10.1371/journal.pone.0170786 (PMC5283650; doi:10.1371/journal.pone.0170786)
Supplement: S2 Text — (DOCX) [file pone.0170786.s007.docx]

Here we provide additional information for readers interested in using the present method.

**Promoting a natural interaction.** For a natural interaction to take place we recommend the following considerations: 1) Choose a space that is comfortable and which defines the boundaries so that the participants don't move out of frame. 2) Avoid screens in which the participants can see themselves during the conversations.

**Interaction center point.** Marking the mutual center point that you expect participants to keep using any marker/sign in the room. Establish such an anchoring point will help participants avoid occlusions throughout the interaction.

**Time synchronization.** Prior to leaving the room for the interaction to take place, ask participants to do a "high-five", which can be used later to synchronize the data from the two cameras. The "high-five" also assists in creating a friendly atmosphere at the start of the interaction.

**Reliable spatial calibration.** To calibrate the spatial coordinates of the cameras, dedicate a short recording using the setup prior or post every session. We used a single person standing still in the interaction center-point facing the middle point between the two cameras while stretching hands sideways. This pose was maintained for a few seconds and allowed reliable calibration.

**Session duration.** Avoid conducting sessions that exceed 45 min as this becomes unnatural and annoying for the standing subjects.
